# Identification, Validation, and Functional Annotations of Genome-Wide Profile Variation between Melanocytic Nevus and Malignant Melanoma

**DOI:** 10.1155/2020/1840415

**Published:** 2020-08-31

**Authors:** Wei Han, Wen-Hao Xu, Jian-Xiong Wang, Jia-Min Hou, Hai-Liang Zhang, Xiao-Yu Zhao, Guo-Liang Shen

**Affiliations:** ^1^Department of Burn and Plastic Surgery, The First Affiliated Hospital of Soochow University, Suzhou 215000, China; ^2^Department of Surgery, Soochow University, Suzhou 215000, China; ^3^Department of Urology, Fudan University Shanghai Cancer Center, Shanghai 200032, China; ^4^Department of Oncology, Shanghai Medical College, Fudan University, Shanghai 200032, China

## Abstract

Cutaneous melanoma (CM) is known as an aggressive malignant cancer; some of which are directly derived from melanocytic nevi, which have been attracting growing attention from the last decades. This study focused on comprehensive identification, validation, and functional annotations of prognostic differentially expressed genes (DEGs) between melanocytic nevus and malignant melanoma in genome-wide profiles. DEGs were obtained using three chip datasets from GEO database to identify after standardization annotation. A total of 73 DEGs were identified as possible candidate prognostic biomarkers between melanocytic nevus and malignant melanoma. In addition, survival curves indicated that six hub genes, including *FABP5*, *IVL*, *KRT6A*, *KRT15*, *KRT16*, and *TIMP2*, were significant prognostic signatures for CM and of significant value to predict transformation from nevi to melanoma. Furthermore, immunohistochemistry staining was performed to validate differential expression levels and prognostic implications of six hub genes between CM tissue and nevus tissues from the First Affiliated Hospital of Soochow University cohort. It suggested that significantly elevated *FABP5*, *IVL*, *KRT6A*, *KRT15*, *KRT16*, and *TIMP2* proteins expressed in the CM than in the nevus tissues. Functional enrichment and significant pathways of the six significant hub genes indicated that the mostly involved hallmarks include the *P53* pathway, *K-ras* signaling, estrogen response late, and estrogen response early. In summary, this study identified significant DEGs participating in the process of malignant transformation from nevus to melanoma tissues based on comprehensive genomic profiles. Transcription profiles of *FABP5*, *IVL*, *KRT6A*, *KRT15*, *KRT16*, and *TIMP2* provided clues of prognostic implications, which might help us evaluate malignant potential of nevus and underlying carcinogenesis progress from melanocytic nevus to melanoma.

## 1. Introduction

Cutaneous melanoma (CM) is an aggressive tumor that is the fifth and sixth most common malignant tumor of men and women, respectively [1]. Worldwide, cutaneous melanoma accounts for 55,500 cancer deaths (0.7% mortality rate) every year [2]. Further, an elevated incidence and aggressive prognosis of melanoma are associated with the metastatic phase, accounting for the 15% 5-year survival rate [3]. The preferred treatment regimen for melanoma is surgical resection of the primary tumor, while metastatic melanoma is much more difficult to treat with radiotherapy and chemotherapy [4]. Recently developed immunotherapies and targeted therapies show promise for improving the prognosis of patients with advanced melanoma [5]. Identification of melanoma-associated oncogenes informs different therapeutic strategies, and small molecule inhibitors are available to target specific proteins involved in the pathogenesis of melanoma [3]. For example, the outcome of a phase II randomized clinical trial suggests that the BRAF inhibitor vemurafenib prolongs the survival of patients with advanced melanoma carrying the *BRAF^V600E^* mutation [6].

Melanocytic nevus, the most common benign skin tumor of humans, is caused by an increase in melanocyte populations in the epidermis and dermis [7]. The growth of melanocyte nevus is regulated by genetic profiles. For example, evidence indicates that clonal melanocyte tumors are triggered by specific oncogenic mutations in components of the mitogen-activated protein kinase (MAPK) signal transduction pathway, most commonly the *BRAF^V600E^* that constitutively activates the protein kinase activity of BRAF [8, 9]. According to the Clark model, the pathogenesis of melanoma assumes that numerous steps are required for the progression from melanocytes to malignant melanoma [10], including formation of banal nevi, dysplastic nevi, melanoma in situ, and invasive melanoma.

Approximately 25% of cutaneous melanomas arise from nevi [11], which may approach 50% in potentially high-risk patients with numerous nevi [12]. Also, ultraviolet radiation will increase melanoma risk, especially among persons with a high nevus count [9]. In melanomas arise from preexisting nevi, the residual original nevi are usually histologically apparent [7]. The results of genetic analysis of benign nevus-melanoma pairs that are consistent with histological findings support the hypothesis that melanoma cells are directly derived from nevus cells [9, 13]. Thus, there is an urgent need to understand the molecular mechanisms involved in the pathogenesis, progression, and recurrence of melanomas. Such efforts are enhanced through the identification of genes that are differentially expressed (DEGs) in melanocytic nevus vs. malignant melanoma. This information will help guide the development of precise diagnostic and therapeutic strategies.

As a rapidly emerging discipline, bioinformatics studies the collection, processing, storage, dissemination, analysis, and interpretation of biological information, to integrate life sciences and computer sciences. Bioinformatics mainly focuses on genomics and proteomics to identify genotypes and phenotypes associated with immune infiltration, tumorigenesis, and progression of melanoma to guide the development of targeted therapy [14]. Here, we analyzed three mRNA microarray datasets from the Gene Expression Omnibus (GEO) data repository to distinguish between melanocytic nevi and melanomas. Subsequently, we performed functional pathway enrichment analysis to further identify the mechanisms underlying malignant transformation. Protein-protein interaction (PPI) network analysis revealed the specific functions of the proteins to evaluate the importance of their interactions potentially associated with the malignant phenotype [15–17]. Furthermore, immunohistochemistry staining was performed to validate differential expression levels and prognostic implications of six hub genes between CM tissue and nevus tissues from the First Affiliated Hospital of Soochow University cohort.

Here, we focused on determining gene expression profiles to identify candidate diagnostic and prognostic biomarkers that may improve the treatment of patients with melanoma as well as illuminate the underlying biological interaction networks. Our findings led us to hypothesize that six significant hub genes, which may contribute to oncogenic activity, are significantly associated with poor prognosis of melanoma. These findings will facilitate efforts to develop new prognostic markers and therapeutic targets that distinguish high-risk nevi.

## 2. Materials and Methods

### 2.1. Patients and Variables

A total of 31 nevus and 31 CM tissues were obtained from 62 patients at the Department of Burn and Plastic Surgery, the First Affiliated Hospital of Soochow University (FAHSU, Suzhou, China) from March 2016 to August 2019. None of the patients had received radiotherapy or chemotherapy before operation. Tissue samples, including nevus and melanoma tissue, were collected during surgery and fixed in 4% paraformaldehyde, available from FAHSU tissue bank. Clinical data was available to obtain from hospital records. This research was supported by the Independent Ethics Committee (IEC) of the FAHSU, and all patients were well informed of storing and upcoming use of their resected specimens for further research purposes.

### 2.2. Acquisition and Standardization of Raw Microarray Dataset

mRNA microarray datasets were screened and obtained from GEO (http://www.ncbi.nlm.nih.gov/geo) [18] testing mRNA expression in melanoma and nevus patients including three chip datasets GSE3189 [19], GSE12391 [20], and GSE46517 [21] (18 nevus and 45 melanoma samples in GSE3189, 18 nevus and 23 melanoma samples in GSE12391, and 9 nevus and 31 melanoma samples in GSE46517) in Affymetrix GPL96 platform. Significant DEGs distinguishing between melanoma and nevus tissues were identified using the limma R package across background correction of probe annotations. Adjusted *p* values (adj. *p*), false discovery rate (FDR), and fold change were used for filtering of DEGs and applying to balancing statistically algorithm. ∣log_2_FC(fold change) | >1.00 and adj. *p* value < 0.01 were considered of statistical significance.

### 2.3. Functional Enrichment of DEGs

The Database for Annotation, Visualization, and Integrated Discovery (DAVID, https://david.ncifcrf.gov/) was utilized to perform functional and pathway enrichment analysis. It can provide systematic and integrative functional annotation tools for users to unravel biological meaning behind the list of genes. Gene ontology (GO) analysis including the biological process (BP), cellular component (CC), and molecular function (MF) and Kyoto Encyclopedia of Genes and Genomes (KEGG) pathway enrichment analysis were conducted for the selected DEGs by DAVID [22, 23]. *p* value < 0.05 was considered statistically significant.

### 2.4. PPI Network Construction and Analysis

In this study, STRING (http://string-db.org; version 11.0) was used to describe protein coregulation of DEGs and measure functional interactions among nodes [24]. The interaction specificity score > 0.4 (the default threshold in the STRING database) was considered statistically significant.

Cytoscape (version 3.5) was utilized to visualize interaction networks obtained from STRING [25]. MCODE (version 1.4.2) of Cytoscape is a plug-in to cluster a given network to identify densely connected regions based on topology [26]. It was utilized to find the most related module network with selection threshold as follows: MCODE scores > 5, degree cut‐off = 2, node score cut‐off = 0.2, Max depth = 100 , and *k* score = 2. The hub nodes of network with connectivity degrees > 10 were selected. Network of 10 genes and neighbor nodes were obtained using cBioPortal (http://www.cbioportal.org/) tool [27]. GO: BP, CC, MF, and KEGG functional enrichment were analyzed and plotted using ClueGO (version 2.5.3) and CluePedia (version 1.5.3) [28].

### 2.5. TCGA Database

A total of 481 CM patients with clinical profiles, among which 475 CM patients with available RNA sequence data downloaded from UCXC (https://xenabrowser.net/datapages/) [29], were consecutively recruited in analyses. Phenotype and transcriptional expression profiles in 481 melanoma patients from TCGA were analyzed and displayed. Clinical and pathological parameters of the cohort were summarized. The 6 prognostic hub genes were identified as dichotomous variables with the median expression.

Gene Expression Profiling Interactive Analysis (GEPIA, http://gepia.cancer-pku.cn/) is a web tool that can provide fast and customizable functionalities based on data from The Cancer Genome Atlas (TCGA; https://tcga-data.nci.nih.gov/tcga/) and the Genotype-Tissue Expression project (GTEx; https://www.gtexportal.org/home/index.html) [30]. GEPIA performs survival analysis based on gene expression levels, using log-rank test for the hypothesis evaluation. The horizontal axis (*x*-axis) represented time in days, and the vertical axis (*y*-axis) showed the probability of surviving or the proportion of people surviving. The dotted lines represented the 95% confidence interval information in the survival plots, with high expression marked in red and low expression marked in blue. The lines presented survival curves of two groups.

Multivariate analysis was applied with Cox regression models using BACK-LR methods to identify the variables, including Clark level (ref. I–III), pT stage (ref. T1–T2), pN stage (ref. N0), pM stage (ref. M0), pathological stage (ref. I–II), FABP5 expression (ref. Low), IVL expression (ref. Low), KRT6A expression (ref. Low), KRT15 expression (ref. Low), KRT16 expression (ref. Low), TIMP2 expression (ref. Low), and two known prognostic biomarkers of CM: S100B expression (ref. Low) and WNT5A expression (ref. Low). *p* values less than 0.05 were considered significant in all tests.

### 2.6. Immunohistochemistry (IHC)

Protein expression levels of significant six hub genes were measured using IHC staining and mouse monoclonal anti-FABP5 antibody (ab84028), anti-involucrin antibody [SY5] (ab68), anti-cytokeratin 6 antibody [Ks6.KA12] (ab18586), anti-cytokeratin 15 antibody [EPR1614Y] (ab52816), anti-cytokeratin 16/K16 antibody [EP1615Y] (ab76416), and anti-TIMP2 antibody (ab180630). Positive or negative staining of a certain protein in one FFPE slide was independently assessed by two experienced pathologists and supervised by a clinician. Based on the staining intensity level (no staining, weak, moderate, and strong staining), the score was ranging from 0 to 3, as previously described [31]. The staining extent was graded from 0 to 4 for the coverage percentage of immunoreactive tumor cells (0%, 1–25%, 26–50%, 51–75%, 76–100%). The overall IHC score grading from 0 to 12 was evaluated according to the multiply of the staining intensity and extent score. Negative staining represented grade 0 to 4 and positive staining from 5 to 12 for each sample.

### 2.7. Transcription Factor Network and Data Processing of Gene Set Enrichment Analysis (GSEA)

Transcription factor regulation networks were constructed in *FABP5*, *IVL*, *KRT6A*, *KRT15*, *KRT16*, and *TIMP2* using R software (version 3.3.2). Significant nodes involved in coregulation of *FABP5*, *IVL*, *KRT6A*, *KRT15*, *KRT16*, and *TIMP2* were described in circle plots (including transcription factor regulation-DNA binding, transcription factor regulation-activation, related lncRNA, targeted miRNA, and protein-protein interaction). Based on data from the TCGA database, GSEA tool (version 2.10.1 package) was used to predict associated up- and downregulated genes and their significantly involved hallmark pathways [32]. For each separate analysis, Student's *t*-test statistical score was performed in consistent pathways and the mean of the differential expression genes was calculated. A permutation test with 1000 times was used to identify the significantly changed pathways. The adj. *p* using the Benjamini and Hochberg (BH) false discovery rate (FDR) method by default was applied to correct for the occurrence of false positive results. Significant related genes were defined with an adj. *p* less than 0.01 and FDR less than 0.25.

## 3. Results

### 3.1. Clinical and Pathologic Characteristics Baseline of CM Patients from TCGA and FAHSU

481 CM patients were enrolled from TCGA cohort and 31 from FAHSU cohort. Clinicopathological parameters of both nevus and CM patients from two cohorts, including age at surgery, gender, location, Clark level, Breslow's depth, TNM stage, and pathologic stage, are shown in Table 1.

### 3.2. Screening and Identification of DEGs between Melanoma and Nevus

**Figure 1:**
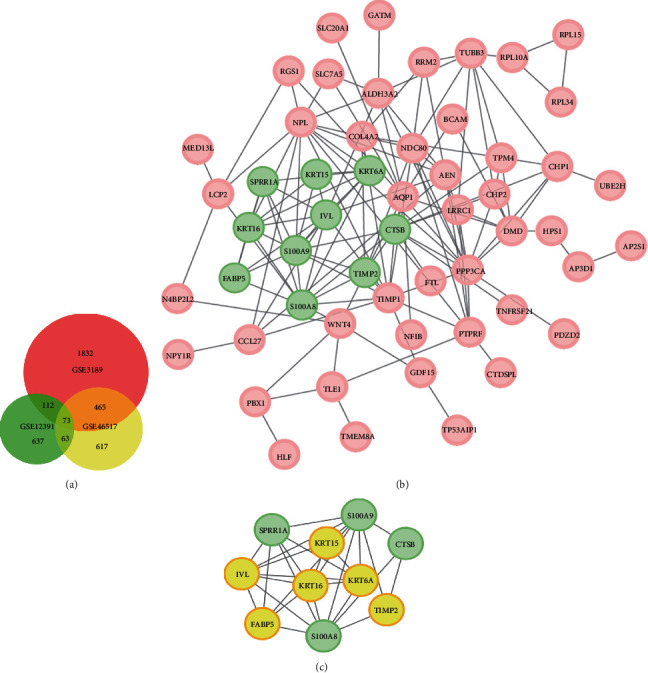
Venn diagram, PPI network, and the most significant module of DEGs. DEGs were selected with ∣log_2_FC | >1 and adj. *p* value < 0.01 among the mRNA expression profiling chip datasets GSE3189, GSE12391, and GSE46517. (a) The three datasets showed an overlap of 73 genes in Venn diagram. (b) The PPI network of DEGs was constructed by using Cytoscape. (c) The most significant module was obtained from PPI network with 10 nodes.

**Figure 2:**
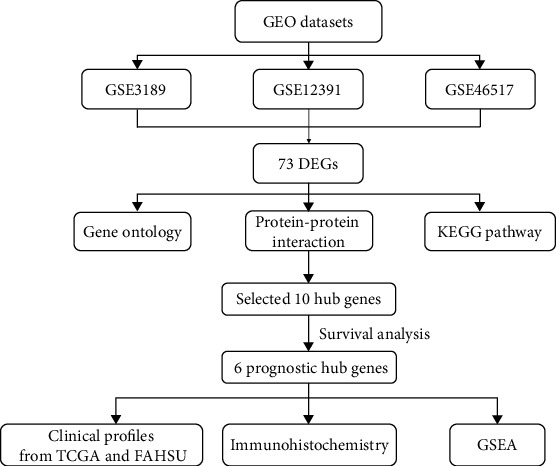
Flowchart of bioinformatics analysis. DEGs: differentially expressed genes; KEGG: Kyoto Encyclopedia of Genes and Genomes; GSEA: gene set enrichment analysis.

DEGs (2,482 in GSE3189, 885 in GSE12391, and 1,218 in GSE46571) were selected after screening and standardization by using the limma package of R. Among the three datasets, 73 genes were overlapped in the Venn algorithm (Figure 1(a)) (Supplementary Table 1) between melanocytic nevus and malignant melanoma. The flowchart is presented in Figure 2.

### 3.3. Functional Enrichment Assessment

As shown in Supplementary Figure 1, GO analysis indicated that changes in biologic processes significantly enriched in ectoderm development, epidermis development, negative regulation of signal transduction, and negative regulation of cell communication. Changes in cellular components were mainly enriched in the non-membrane-bounded organelle and intracellular non-membrane-bounded organelle. Function annotations were mostly enriched in structural molecule activity and structural constituent of cytoskeleton.

### 3.4. PPI Network Establishment

We constructed the coregulated network of DEGs (Figure 1(b)) and subsequently found the most significant module panel by using plug-in MCODE of Cytoscape (Figure 1(c)). With DAVID functional analysis of 10 hub genes, enrichment profiles suggested that hub genes in this module were primarily enriched in ectoderm development, epidermis development, structural molecule activity, structural constituent of cytoskeleton, and cytoskeleton (Table 2).

### 3.5. Hub Gene Selection and Analysis

**Figure 3:**
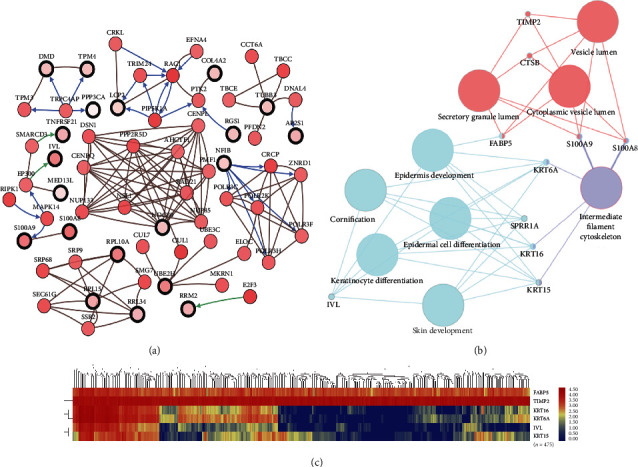
Interaction network and biological process analysis of the hub genes. (a) Hub genes and their coexpression network were analyzed by using cBioPortal. Nodes with bold black outline represent hub genes. Nodes with thin black outline represent the coexpression genes. (b) The biological process analysis of hub genes was constructed by using ClueGO. Different colors of nodes refer to the functional annotation of ontologies. Corrected *p* value < 0.01 was considered statistically significant. (c) Hierarchical partitioning of 6 significant hub genes was obtained from DNA microarrays (*n* = 475). It represented the level of expression of 6 genes across a number of comparable samples with high expression samples marked in red and low in blue.

After statistical selection, *SPRP1A*, *S100A8*, *S100A9*, *CTSB*, *FABP5*, *IVL*, *KRT6A*, *KRT15*, *KRT16*, and *TIMP2* were verified as hub genes. Transcriptional coregulation of the ten hub genes and their neighbor nodes was set up visually (Figure 3(a)). GO: BP and KEGG functional annotation is displayed in Figure 3(b), with detailed function annotations listed in pie charts (Supplementary Figure 2). 55.56% terms belonged to cornification, 33.33% to vesicle lumen, and 11.11% to intermediate filament cytoskeleton. Heat map, based on TCGA cohort (*n* = 475), showed that potential coexpression relationship may be found in the 6 significant hub genes, which could suggest basic value in prognostic prediction (Figure 3(c)).

### 3.6. Clinicopathological Statistical Analysis and Survival Outcomes in CM Patients from TCGA

**Figure 4:**
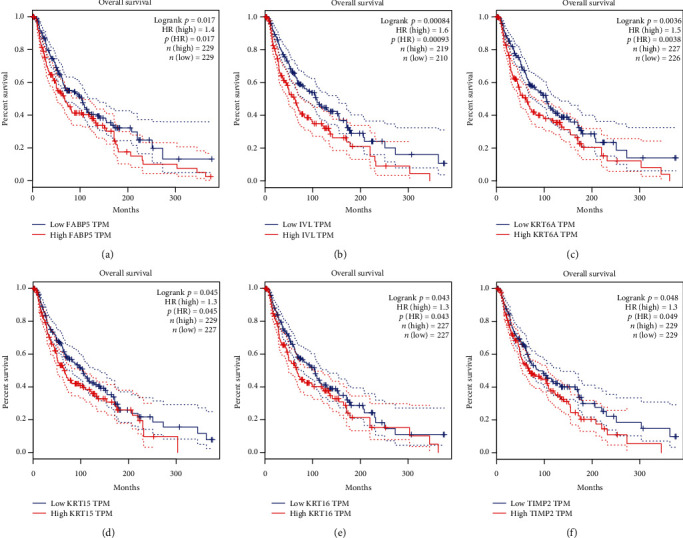
Univariate survival analysis of the hub genes was performed using the Kaplan-Meier curve. The horizontal axis (*x*-axis) represented time in days, and the vertical axis (*y*-axis) showed the probability of surviving or the proportion of people surviving. The dotted lines represented the 95% confidence interval information in the survival plot, with high expression marked in red and low expression marked in blue. The lines presented survival curves of two groups. Each elevated expression in 6 hub genes showed markedly significant worse OS in melanoma samples (*p* < 0.05).

Significant survival outcomes (OS: *p* < 0.05) were found in Figures 4(a)–4(f). *FABP5*, *IVL*, *KRT6A*, *KRT15*, *KRT16*, and *TIMP2* expression profiles suggested relatively significant elevated expression in tumor tissues compared with the corresponding normal tissues. In addition, multivariate Cox regression analysis of OS and PFS in TCGA cohort using BACK-LR methods was performed (Supplementary Table 2). In the COX models, we recruited two known prognostic biomarkers of melanoma, S100B, and WNT5A, to better identify the prognostic value of the hub genes. As shown in OS analysis, KRT6A along with S100B and WNT5A was considered statistically significant. FABP5 and S100B showed significant prognostic value in PFS analysis.

**Figure 5:**
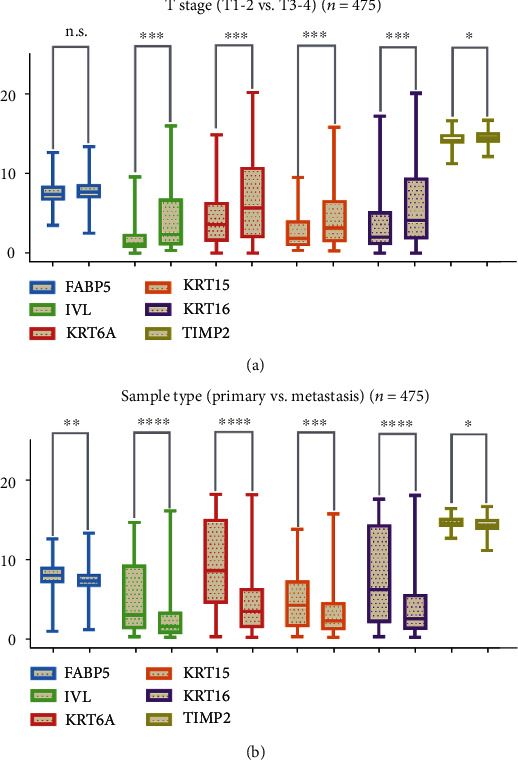
(a) Except FABP5, elevated expression patterns of *IVL*, *KRT6A*, *KRT15*, *KRT16*, and *TIMP2* were significantly associated with T stage (T1-T2 vs. T3-T4) (*n* = 475). (b) Six hub genes are highly expressed in primary than metastatic sites (*n* = 475) (FABP5 presents in light blue, IVL in light green, KRT6A in red, KRT15 in orange, KRT16 in purple, and TIMP2 in dark green).

Except FABP5, elevated expression patterns of *IVL*, *KRT6A*, *KRT15*, *KRT16*, and *TIMP2* were significantly associated with T stage (T1-T2 vs. T3-T4). In addition, six prognostic hub genes are highly expressed in primary than metastatic sites, plotted in Figure 5.

### 3.7. IHC Staining Analyses in 62 Patients from FAHSU Cohort

**Figure 6:**
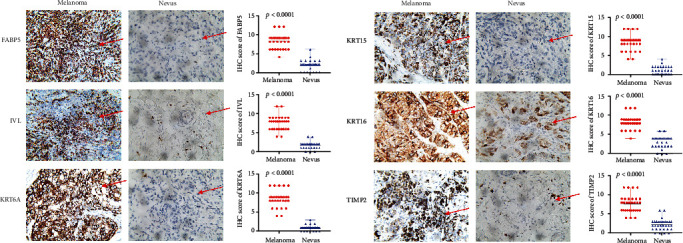
(a) IHC staining indicated significantly elevated FABP5, IVL, KRT6A, KRT15, KRT16, and TIMP2 expressions in terms of density and intensity in melanoma tissues compared with nevus tissues. All melanocyte contents were shown in red arrows. (b) Scatter plots of IHC score between melanoma and nevus tissues were illustrated (*p* < 0.0001).

Next, to validate differential expressions of six prognostic hub genes between CM tissues and nevus tissues, we performed IHC analysis and found significantly elevated *FABP5*, *IVL*, *KRT6A*, *KRT15*, *KRT16*, and *TIMP2* protein expressions in the CM than in the nevus tissues. The results and the scatter plots of IHC score are illustrated in Figure 6.

### 3.8. Significant Genes and Pathway Obtained by GSEA

**Figure 7:**
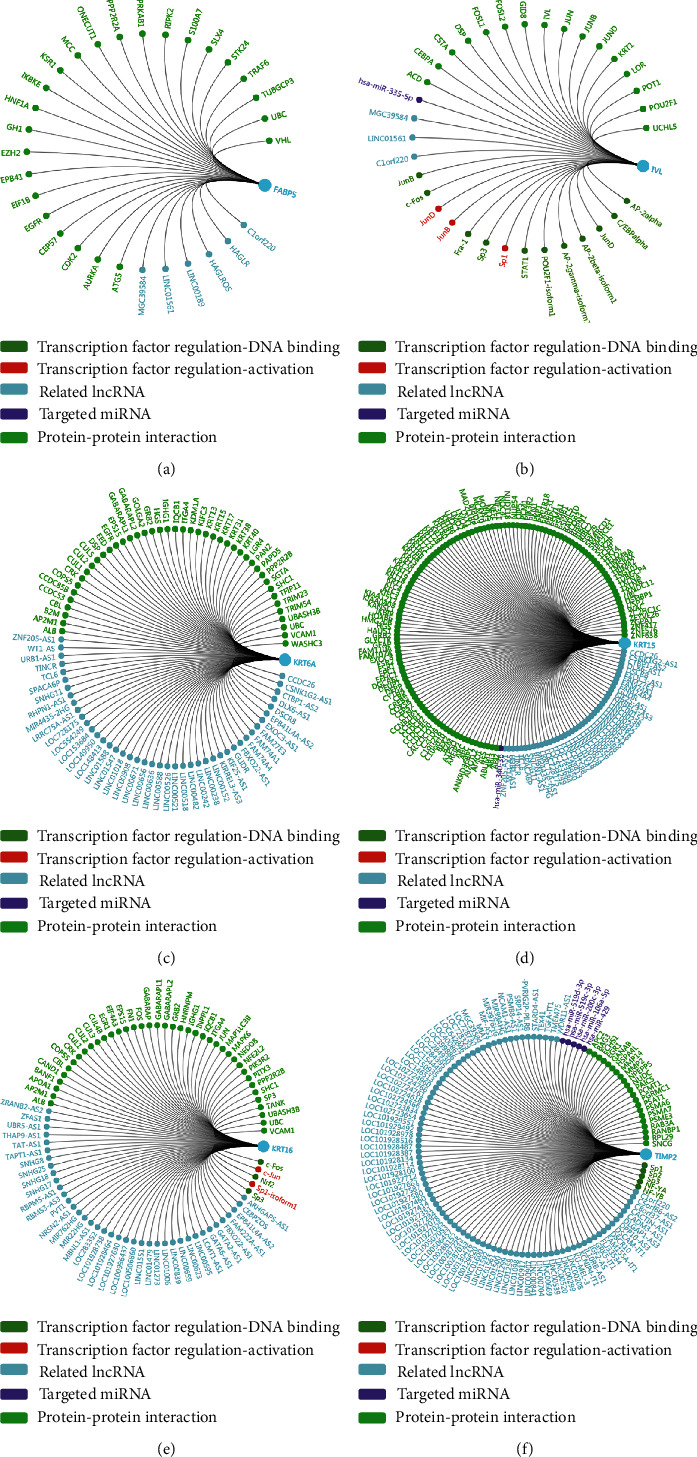
Transcription factor regulation network was constructed in FABP5 (a), IVL (b), KRT6A (c), KRT15 (d), KRT16 (e), and TIMP2 (f). Significant nodes were marked in different colors in line with hub genes (transcription factor regulation-DNA binding, transcription factor regulation-activation, related lncRNA, targeted miRNA, and protein-protein interaction).

**Figure 8:**
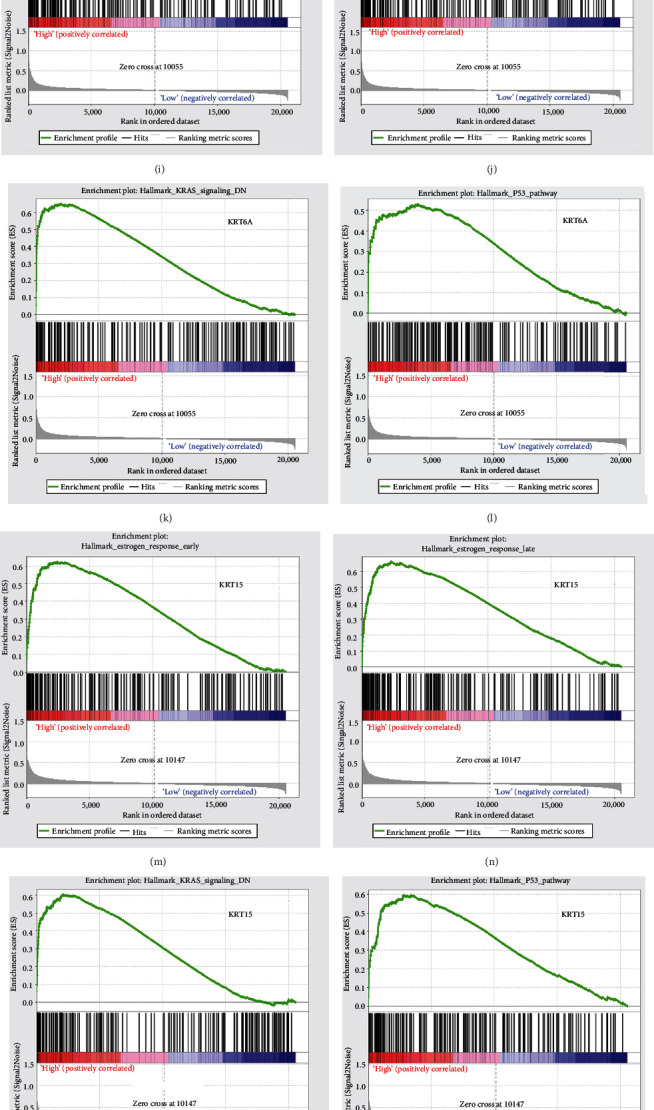
A total of 100 significant genes were obtained from GSEA with positive and negative correlations. GSEA was used to perform hallmark analyses in *FABP5*, *IVL*, *KRT6A*, *KRT15*, *KRT16*, and *TIMP2*, respectively. The most involved significant pathways included the P53 pathway, estrogen response late, estrogen response early, and K-ras signaling.

Transcriptional regulation networks among *FABP5*, *IVL*, *KRT6A*, *KRT15*, *KRT16*, and *TIMP2* are displayed in Figure 7. Significantly involved nodes (including transcription factor regulation-DNA binding, transcription factor regulation-activation, related lncRNA, targeted miRNA, and protein-protein interaction) were marked in different colors. Subsequently, a total of 100 significant genes were obtained from GSEA, and the genes with positive correlation were plotted. GSEA analysis, including *FABP5*, *IVL*, *KRT6A*, *KRT15*, *KRT16*, and *TIMP2*, suggested that the most involved hallmarks included the *P53* pathway, *K-ras* signaling, estrogen response late, and estrogen response early. The details are illustrated in Figure 8.

## 4. Discussion

Melanoma is an aggressive and devastating cancer directly derived from melanocytic nevus, characterized by a typically high incidence in people with the *BRAF^V600E/K^* mutation [33]. Alterations in the activity of the MAPK signal transduction pathway are associated with metastasis in melanoma patients [34, 35]. Specifically, constitutively activated components of the MAPK pathway serve as targets for therapy of melanoma [5]. However, available evidence is insufficient for developing management or diagnostic strategies to improve the survival of patients with melanoma. For example, a study of the expressions of *PD-L1*, *PD-L2*, *PD-1*, and *CYT* in melanomas found that mutation density contributes significant prognostic value [36]. Unfortunately, most patients with melanoma, which are initially diagnosed with highly aggressive and progressive disease, are therefore not candidates for curative therapies [2]. Hence, highly effective biomarkers for diagnosis and treatment are urgently required.

Aberrant genetic and epigenetic regulation of key metabolic pathways is known to contribute towards the development and progression of CM. Here, our analysis of the expression profiles of melanocytic nevus and melanomas identified 73 DEGs and 10 hub genes, as well as the functional enrichment and significant pathways. GO and KEGG enrichment indicated significant associations with the terms ectoderm development, epidermis development, negative regulation of signal transduction, and negative regulation of cell communication. In addition, GSEA analysis showed that the most involved hallmarks included the *P53* pathway, *K-ras* signaling, estrogen response late, and estrogen response early. Increasing evidence supports a role for *P53* in the progression of melanoma, particularly in NRAS-driven melanomas, which grow more aggressively compared with those with *BRAF* mutations [37]. P53 mutations occur in 15% of melanomas with *NRAS* mutations [37]. We suggest therefore that reactivating P53 expression in melanoma may be potentially important for enhancing therapy. Constitutive activation of KRAS stimulates cell proliferation and inhibits apoptosis. Increasing evidence showed that KRAS mutations are closely associated with multiple cancers, including non-small-cell lung cancer, colorectal cancer, and pancreatic cancer, and NRAS mutations are present in melanomas [38]. Therefore, role of KRAS signaling in melanoma must be evaluated. Moreover, evidence shows significant protective roles for estrogen signaling in the pathogenesis and progression of melanoma [39]. Although the mechanism is unknown, this clinical association suggests the importance of estrogen signaling in the progression of the malignant phenotype of melanoma.

Further, among the 6 prognostic hub genes, differential expressions of the genes encoding fatty acid-binding protein 5 (*FABP5*); involucrin (*IVL*); keratins 6A, 15, and 16 (*KRT6A*, *KRT15*, and *KRT16*); and tissue inhibitor of metalloproteinases 2 (*TIMP2*) was significantly associated with prognosis of patients with melanoma based on the survival analysis and COX models.

FABP5 plays an important role in binding free fatty acids, as well as in the regulation of lipid metabolism and transport. Urinary excretion of FABP5 is detected in patients with stages II and III cutaneous melanoma but not those with stage IV melanoma [40]. Insufficient information is available concerning the role of FABP5 in melanomas vs. its association with prognosis [41, 42]. In the present study, FABP5 was found with significant prognostic value not only in survival analysis by GEPIA but also in multivariate Cox models by BACK-LR methods. Hence, further research is required to confirm our hypothesis.

IVL is a component of the human skin that contributes to the formation of the envelope that protects corneocytes [43]. IVL is produced in a free form during the early stages of the terminal differentiation of keratinocytes. Increased expression of IVL correlates with undesirable outcomes of squamous cell carcinoma [44], and IVL contributes to inflammatory skin diseases such as psoriasis [45]. Our present findings therefore likely will encourage further investigations of the clinical significance of IVL in human disease, although few reports address its value for predicting the prognosis of melanoma.

Keratin (KRT), which is a constituent of the hair, nails, and the outer layer of the human skin, protects epithelial cells from damage or pressure [46]. The gene encoding KRT6A, which is located within the type II keratin gene cluster on human chromosome 12q [47], is closely associated with the prognosis and diagnosis of lung and breast cancers; however, few studies address its role in melanoma [48, 49]. In our study, KRT6A showed significant prognostic value among all the hub genes according to the multivariate Cox models by BACK-LR methods, indicating it might act unique role in CM patients' survival outcomes. The type I cytokeratin KRT15 is expressed by certain progenitor basal cells within the epithelium [50]. For example, KRT15 is present in the adult and fetal human skin of hair-bearing, non-hair-bearing, and palmoplantar regions and is coordinately expressed with melanoma-associated chondroitin sulfate proteoglycan [51]. The type I cytokeratin KRT16 forms a complex with KRT6 in numerous epithelial tissues. Mutations in KRT6 are associated with hereditary skin diseases, and KRT16 contributes to the immune response to tumors and in tumor cell development [52]. Moreover, the levels of KRT16 expression may discriminate metastasis from primary melanoma [53]. These findings demonstrate the significant role of keratins in melanoma.

Evidence indicates that TIMP2 functions as a suppressor of metastasis. For example, elevated expression of TIMP2 suppresses the proliferation of melanoma cells via the Wnt/𝛽-catenin signal transduction pathway, indicating that TIMP2 contributes to the pathogenesis and progression of melanoma [54]. In the current study, TIMP2 was found elevated in primary CM, which is consistent with previous studies. Further studies are needed to elucidate underlying potential carcinogenesis progressive nevus and melanoma.

This study is the first to our knowledge that has attempted to construct a gene regulatory network incorporating DEGs between nevus and melanoma, as well as to functionally annotate hub genes in melanoma. Further, alterations in the expression profiles of *FABP5*, *IVL*, *KRT6A*, *KRT15*, *KRT16*, and *TIMP2* were significantly associated with worse prognosis, indicating that these hub genes may participate in the aggressive transformation from a nevus to malignant melanoma. While merely expression level of hub genes was identified in this study, thus further functional works, as well as validated cohorts, were needed to verify the absoluteness of these findings.

## 5. Conclusion

In summary, this study identified significant DEGs participating in the process of malignant transformation from nevus to melanoma tissues. Expression profiles of *FABP5*, *IVL*, *KRT6A*, *KRT15*, *KRT16*, and *TIMP2* provide clues of prognostic implications, which might help us evaluate malignant potential of nevus and underlying carcinogenesis progress from melanocytic nevus to melanoma. These hub genes are associated with major biological pathways such as the *P53* pathway, *K-ras* signaling, estrogen response late, and estrogen response early. However, further studies are required to elucidate molecular pathogenesis and alteration in signaling pathways of these hub genes in melanoma.

## Figures and Tables

**Table 1:** Clinicopathological characteristics of the patients from two cohorts.

Characteristics	FAHSU cohort		TCGA cohort (*N* = 481)
	Nevi (*N* = 31)	Melanoma (*N* = 31)	
*N* (%)			
Age			
≤60 years	23 (74.2)	13 (41.9)	258 (54.7)
>60 years	8 (25.8)	18 (58.1)	214 (45.3)
Gender			
Male	14 (45.2)	20 (64.5)	297 (61.9)
Female	17 (54.8)	11 (35.5)	183 (38.1)
Location			
Extremity	21 (67.7)	24 (77.4)	—
Trunk	10 (32.3)	7 (22.6)	—
Clark level			
I	—	13 (41.9)	6 (1.8)
II	—	15 (48.4)	18 (5.5)
III–IV	—	3 (9.7)	246 (75.5)
V	—	0 (0)	56 (17.2)
Breslow's depth (mm)			
≤0.75	—	5 (16.1)	36 (10.2)
0.76-1.50	—	10 (32.2)	65 (18.4)
1.51-4.00	—	13 (41.9)	106 (30.0)
>4.00	—	3 (9.8)	146 (41.4)
pT stage			
T1-T2	—	19 (61.3)	121 (32.7)
T3-T4	—	12 (38.7)	249 (67.3)
pN stage			
N0	—	31 (100)	236 (65.0)
N1	—	0 (0)	75 (20.7)
N2	—	0 (0)	52 (14.3)
pM stage			
M0	—	31 (100)	424 (94.4)
M1	—	0 (0)	25 (5.6)
Pathologic stage			
I-II	—	31 (100)	233 (53.9)
III-IV	—	0 (0)	199 (46.1)
Persistent distant metastasis			
No	—	31 (100)	217 (46.1)
Yes	—	0 (0)	254 (53.9)
Cell atypia/atypical hyperplasia			
No	31 (100)	—	—
Yes	0 (0)	—	—

FAHSU: the First Affiliated Hospital of Soochow University; TCGA: The Cancer Genome Atlas.

**Table 2:** GO enrichment analysis of DEGs in the most significant module.

Term	Description	Count in gene set	*p* value
GO:0007398	Ectoderm development	6	7.87*E* − 08
GO:0008544	Epidermis development	5	3.96*E* − 06
GO:0005198	Structural molecule activity	5	5.83*E* − 04
GO:0005200	Structural constituent of cytoskeleton	3	1.12*E* − 03
GO:0005856	Cytoskeleton	5	3.62*E* − 03
GO:0005882	Intermediate filament	3	4.08*E* − 03
GO:0045111	Intermediate filament cytoskeleton	3	4.26*E* − 03
GO:0043228	Non-membrane-bounded organelle	6	5.00*E* − 03
GO:0043232	Intracellular non-membrane-bounded organelle	6	5.00*E* − 03
GO:0001533	Cornified envelope	2	1.20*E* − 02
GO:0018149	Peptide cross-linking	2	1.71*E* − 02
GO:0031424	Keratinization	2	2.82*E* − 02

GO: Gene Ontology; DEGs: differentially expressed genes.

## Data Availability

The datasets analyzed for this study can be found in the GEO (https://www.ncbi.nlm.nih.gov/geo) and TCGA (https://www.cancer.gov).

## Supplementary Materials

Supplementary Materials Supplementary Figure 1: functional and pathway enrichment analyses were performed using DAVID in bubble chart. Supplementary Figure 2: a network of the 10 genes and their coexpression genes was visualized and displayed in detail. Supplementary Table 1: gene symbols of 73 DEGs. Supplementary Table 2: multivariate Cox regression analysis of OS and PFS in TCGA cohort using BACK-LR methods (OS: overall survival; PFS: progression-free survival; TCGA: The Cancer Genome Atlas).
