# Supplementary material for: Identification, Validation, and Functional Annotations of Genome-Wide Profile Variation between Melanocytic Nevus and Malignant Melanoma
Source: Biomed Res Int. 2020 Aug 31;2020:1840415. doi: 10.1155/2020/1840415 (PMC7479462; doi:10.1155/2020/1840415)

Supplementary Figure 1. Functional and pathway enrichment analyses were performed using DAVID in bubble chart. (A)Changes in biologic processes of DEGs were mainly enriched in the ectoderm development, epidermis development, negative regulation of signal transduction and negative regulation of cell communication. (B)Changes in cellular components were mostly enriched in non-membrane-bounded organelle and intracellular non-membrane-bounded organelle. (C)GO analysis results showed that changes in molecular function of DEGs were significantly enriched in structural molecule activity and structural constituent of cytoskeleton.


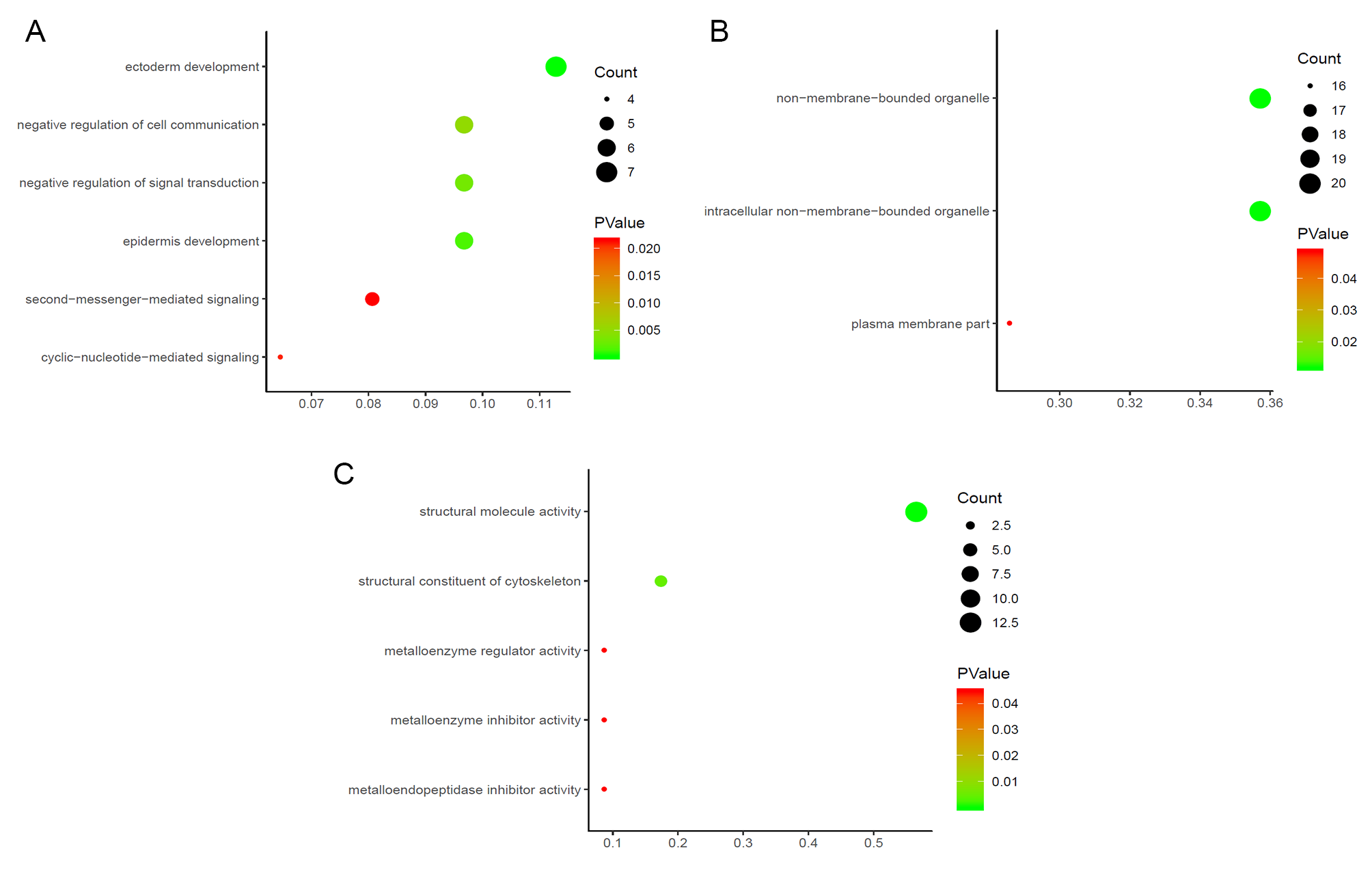


Supplementary Figure 2. A network of the 10 genes and their co-expression genes were visualized and displayed in detail. (A) The BP,CC,MF and KEGG enrichment analyses of the hub genes were shown in different color. (B) The detailed functional notes and classification pie charts are listed as follows. 55.56% terms belong to cornification, 33.33% to vesicle lumen and 11.11% to intermediate filament cytoskeleton.


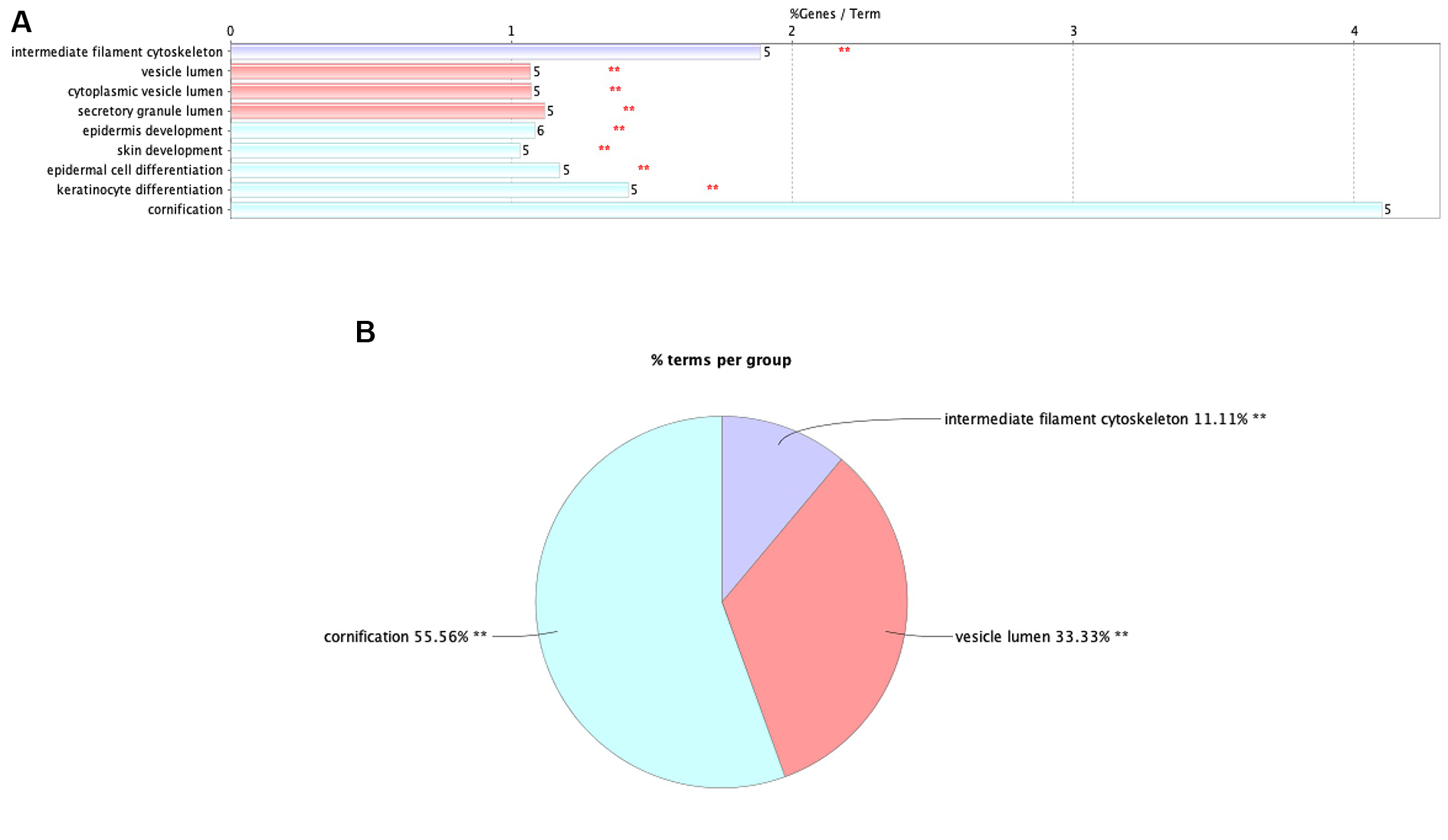

Supplement: Supplementary Materials — Supplementary Figure 1: functional and pathway enrichment analyses were performed using DAVID in bubble chart. Supplementary Figure 2: a network of the 10 genes and their coexpression genes was visualized and displayed in detail. Supplementary Table 1: gene symbols of 73 DEGs. Supplementary Table 2: multivariate Cox regression analysis of OS and PFS in TCGA cohort using BACK-LR methods (OS: overall survival; PFS: progression-free survival; TCGA: The Cancer Genome Atlas). [file 1840415.f1.zip › 1840415.f1.docx]
