# Supplementary material for: Identification, Validation, and Functional Annotations of Genome-Wide Profile Variation between Melanocytic Nevus and Malignant Melanoma
Source: Biomed Res Int. 2020 Aug 31;2020:1840415. doi: 10.1155/2020/1840415 (PMC7479462; doi:10.1155/2020/1840415)
Supplement: Supplementary Materials — Supplementary Figure 1: functional and pathway enrichment analyses were performed using DAVID in bubble chart. Supplementary Figure 2: a network of the 10 genes and their coexpression genes was visualized and displayed in detail. Supplementary Table 1: gene symbols of 73 DEGs. Supplementary Table 2: multivariate Cox regression analysis of OS and PFS in TCGA cohort using BACK-LR methods (OS: overall survival; PFS: progression-free survival; TCGA: The Cancer Genome Atlas). [file 1840415.f1.zip › 1840415.f2.docx]

**Supplementary table 1.** Gene symbols of 73 DEGs.

| **Gene symbol** |
| --- |
| RPL34 |
| RPL10A |
| S100A9 |
| KRT6A |
| DMD |
| NPY1R |
| GATM |
| RRM2 |
| IVL |
| AP3D1 |
| PPP3CA |
| ALDH3A2 |
| NDC80 |
| PTPRF |
| CTSB |
| SPRR1A |
| AQP1 |
| PBX1 |
| TUBB3 |
| TP53AIP1 |
| RGS1 |
| TLE1 |
| S100A8 |
| LRRC1 |
| AEN |
| NPL |
| TPM4 |
| FTL |
| COL4A2 |
| WNT4 |
| KRT16 |
| N4BP2L2 |
| TIMP2 |
| GDF15 |
| CCL27 |
| SLC7A5 |
| MED13L |
| NFIB |
| UBE2H |
| TMEM8A |
| RPL15 |
| AP2S1 |
| CHP1 |
| CHP2 |
| TIMP1 |
| KRT15 |
| HPS1 |
| BCAM |
| HLF |
| TNFRSF21 |
| FABP5 |
| LCP2 |
| PDZD2 |
| SLC20A1 |
| CTDSPL |
| PCDH11Y |
| FCGR3A |
| WIF1 |
| MIR4435-2HG |
| FCGR1B |
| CXCL10 |
| CCL18 |
| GBP5 |
| CXCL9 |
| CXCL8 |
| TFEC |
| PRAME |
| ZNF595 |
| IFITM4P |
| DNAJC15 |
| AHNAK |
| RGMB |
| ARMC9 |

**Supplementary Table 2.** Multivariate Cox regression analysis of OS and PFS in TCGA cohort using BACK-LR methods (OS: overall survival; PFS: progression-free survival; TCGA: The Cancer Genome Atlas)

| Covariates | OS | | |  | PFS | | |
| --- | --- | --- | --- | --- | --- | --- | --- |
|  | HR | 95% CI | *P* value |  | HR | 95% CI | *P* value |
| pT stage (ref. T1-T2) | 1.518 | 1.155-1.994 | **0.003** |  | 2.393 | 1.586-3.611 | **<0.001** |
| pN stage (ref. N0) | 1.712 | 1.293-2.267 | **<0.001** |  | - | - | **-** |
| pM stage (ref. M0) | 2.085 | 1.156-3.768 | **0.015** |  | 2.242 | 1.070-4.702 | **0.033** |
| Pathologic stage (ref. I-II) | - | - | - |  | 1.737 | 1.140-2.647 | **0.010** |
| Clark level (ref. I-III) | 1.181 | 0.982-1.420 | 0.078 |  | - | - | - |
| FABP5 expression | - | - | - |  | 1.204 | 1.046-1.386 | **0.010** |
| IVL expression | - | - | - |  | - | - | **-** |
| KRT6A expression | 1.070 | 1.037-1.103 | **<0.001** |  | - | - | **-** |
| KRT15 expression | - | - | - |  | - | - | **-** |
| KRT16 expression | - | - | - |  | - | - | **-** |
| TIMP2 expression | - | - | - |  | - | - | **-** |
| S100B expression | 0.887 | 0.835-0.943 | **<0.001** |  | 0.883 | 0.812-0.960 | **0.004** |
| WNT5A expression | 0.927 | 0.865-0.994 | **0.034** |  | - | - | **-** |
